# A Rare Case of Chromosomal Abnormality: 19q13.11 Deletion in a Patient With Aplasia Cutis Congenita and Ambiguous Genitalia

**DOI:** 10.7759/cureus.82871

**Published:** 2025-04-23

**Authors:** Anshuman Srivastava, Rishika Sharma, Sandhya Kadam, Archana Reddy Bongurala

**Affiliations:** 1 Family Medicine, Family HealthCare Network, Visalia, USA; 2 Pediatric Medicine, Kaweah Delta Health Care District, Visalia, USA; 3 Pediatrics, Family HealthCare Network, Visalia, USA; 4 Pediatrics, Omni Family Health California, Bakersfield, USA

**Keywords:** aplasia cutis congenita acc, aplasia cutis congenita management, chromosomal abnormality, congenital defects, scalp defects

## Abstract

Aplasia cutis congenita (ACC) is described as the local or generalized absence of the skin, the most common site being the scalp. Approximately one-third to one-sixth of the cases are associated with underlying bone and scalp dura mater defects. ACC may have other congenital defects, some of which are known as cleft deformities, abdominal wall defects, limb anomalies, and fetus papyraceus. Some of the ACC cases involve lesser-known defects. Different treatment options are available without clear, definitive guidelines. This case involves a patient who was born without an antenatal diagnosis of ACC and was found to have ambiguous genitalia and a chromosomal abnormality, a 19q13.11 deletion. Based on available treatment options in the literature, this patient was treated with a multidisciplinary specialty approach with conservative options and is waiting for future surgical repair. Limited cases of ACC are documented in the literature, which are considered great sources for obtaining knowledge about ACC. This case report aims to add to this great source with a discussion of the advantages and disadvantages of available treatment options and possible etiopathological factors. This case underscores the importance of a multidisciplinary approach to managing ACC, given its potential for diverse clinical presentations and the need for individualized treatment plans. ACC can be associated with various anomalies, including skeletal, neurological, and other congenital malformations.

## Introduction

Because skin alterations and genital defects are extremely difficult to detect using ultrasound, few patients are still born with congenital defects. At birth, they can be diagnosed with other associated anomalies. In this case report, the infant was born unexpectedly with a significant defect to his head and was diagnosed with aplasia cutis congenita (ACC) with an underlying chromosomal abnormality, 19q13.11 deletion. ACC is known to be associated with other syndromes involving multiple systems and 19q13.11 deletion syndrome, which includes growth and developmental delay, intellectual disability, dysmorphological features, heart issues, skin, and musculoskeletal anomalies. The causes of ACC are not well defined. It may be attributed to the local or generalized absence of layers of the skin during prenatal skin development stages [1].

Different management options are available. However, there are still no clear guidelines for conservative or surgical options, including plastic surgery. The spontaneous occurrence of ACC and its association with other congenital anomalies are known. A definitive consensus is required for the optimal management of the condition. Data indicate approximately 500 cases, serving as the best sources to study presentation, possible underlying etiopathophysiology, association with other congenital anomalies, and management options [2].

Our case report highlights a rare association with an underlying chromosomal abnormality and ambiguous genitalia, aiming to contribute to the existing body of knowledge on ACC. The presence of ambiguous genitalia and chromosomal abnormalities in this patient underscores the importance of the involvement of an interdisciplinary approach in the management of these patients, including genetics.

## Case presentation

A male infant was born to a 30-year-old woman (G2P1) at 38.4 weeks of gestation via vaginal delivery. The mother had a history of gestational diabetes mellitus (GDM) managed with diet and inadequately treated Group B streptococcus (GBS) infection. Maternal prenatal laboratory results were unremarkable. The infant's birth weight was 2.230 kg, length was 0.450 m, and head circumference was 0.295 m. Apgar scores were 2 and 8 at one and five minutes, respectively.

**Figure 1:**
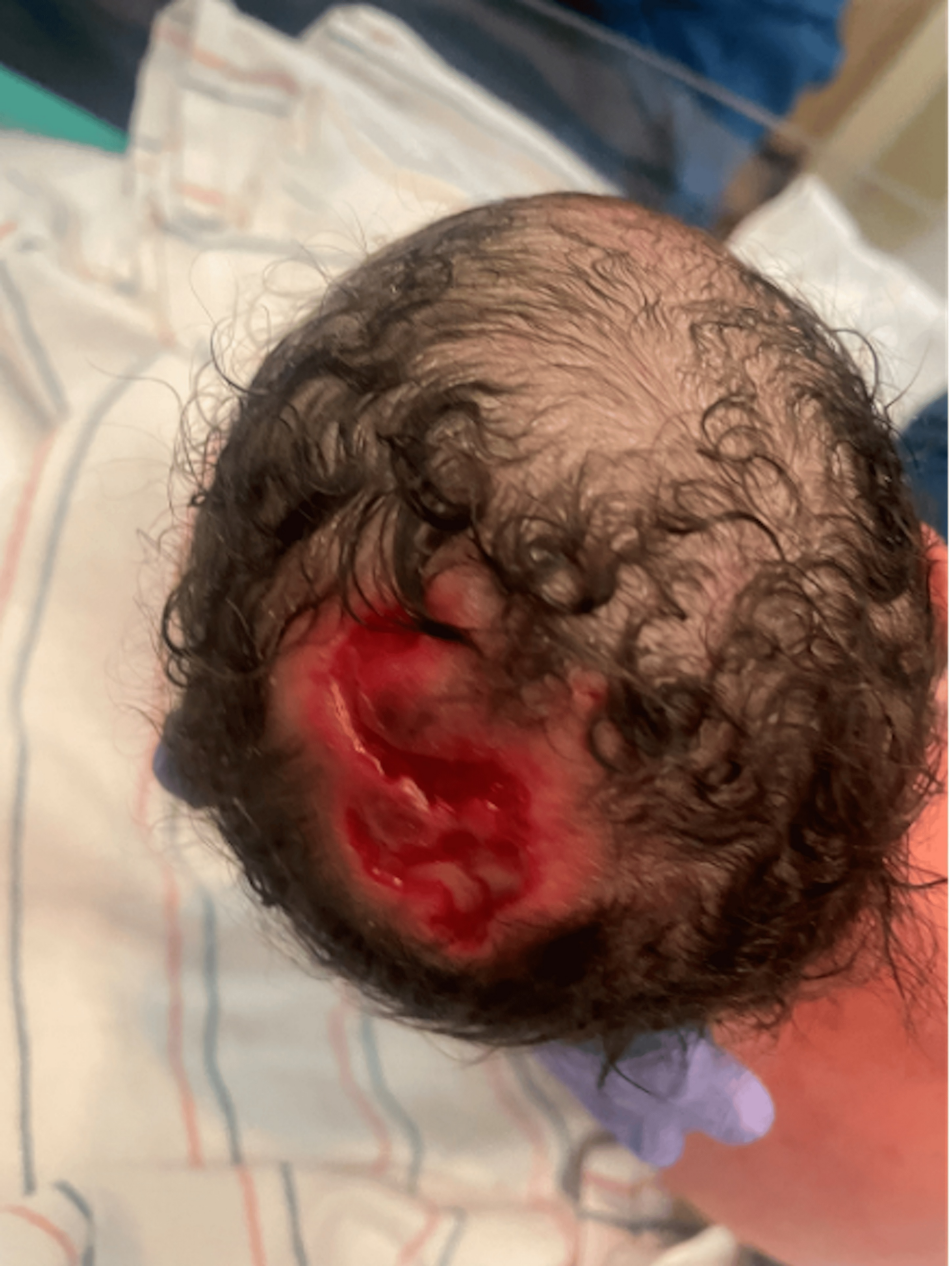
An infant at three hours of age with aplasia cutis congenita

On exam, the patient had an open S-shaped punched-out lesion, a significant scalp defect, covered with granulation tissue on the scalp, and a gray hair patch on the anterior scalp (Figure 1). Additionally, ambiguous genitalia, a microphallus with hypospadias, and bilateral large reducible inguinal hernia were noted on examination. For the differential diagnosis of ambiguous genitalia at birth, such as congenital adrenal hyperplasia, a hormonal workup was done. Lab work showed normal levels of cortisol and 17-hydroxyprogesterone (17OH), undetectable levels of estradiol, and pubertal levels of luteinizing hormone (LH) and follicle-stimulating hormone (FSH) (due to mini-puberty of infancy). Additional labs showed normal dihydrotestosterone, testosterone, and anti-Müllerian hormone (AMH) levels. The examination findings prompted the referral to genetics and evaluation for syndromic association. The features of the patient did not fit into any specific syndrome. However, the microarray finding was significant for a pathogenic deletion of 19q13.11 and showed 46 XY male karyotypes. Further workup included imaging studies. The abdominal ultrasound showed no Müllerian structures, and the renal ultrasound demonstrated no hydronephrosis. The scrotal ultrasound showed testes near the bladder level. MRI of the brain was normal.

To further evaluate the patient, consultations were obtained from pediatric neurosurgery, urology, and endocrinology. Neurosurgery recommended ongoing wound care with the possibility of future surgical intervention. Endocrinology initiated intramuscular testosterone injections monthly for three doses, as recommended by pediatric surgery, to facilitate hypospadias repair. The patient underwent bilateral inguinal hernia repair at eight months of age. Currently, at 14 months of age, the patient awaits hypospadias repair. Neurosurgery continues to recommend future reconstructive surgery for the scalp defect. The patient has been managed with a conservative approach, and the timing of definitive surgical intervention will depend on the healing and closure of the scalp defect.

## Discussion

ACC is an uncommon malformation involving the scalp as the most common site. It also affects other body parts and is associated with other congenital anomalies, adding features of those syndromes to the presentation. Superficial lesions have better outcomes and fewer complications. Spontaneous healing is known to occur. Both conservative management and surgical treatment options are advocated [3].

Pathogenesis

The pathogenesis of ACC is likely multifactorial. During early gestation, rapid fetal brain growth occurs concurrently with hair follicle development and patterning. A primary hypothesis suggests that disruptions in these processes may lead to overlying skin defects. While most ACC cases are sporadic, familial occurrences have been reported, suggesting potential genetic predispositions. Inheritance patterns may include autosomal dominant inheritance with reduced penetrance or autosomal recessive inheritance. Given the possibility of familial occurrence, evaluating other family members for potential skin abnormalities is recommended [4].

Etiology

Based on the available literature, several factors probably contribute to the development of ACC. The exact etiopathophysiology is not known. In utero, mechanical factors and physical trauma to the mother can compromise the vascular blood supply to the fetus. Adhesions formed between the amniotic membrane and fetal skin, the formation of the amniotic band, and the sequence of amniotic fluid rupture can be causative factors. Reabsorption of a dead fetus in a multi-gestational pregnancy leads to vanishing twin syndrome and sometimes compression of the fetus, leading to a parchment state known as fetus papyraceus. ACC is known to be associated with syndromes such as Adams-Oliver syndrome, Cutis Marmorata Telangiectasia syndrome, Bart syndrome, and epidermolysis bullosa [5].

Genetic factors implicated in the development of ACC include mutations in genes such as *BMS1* and *UBA2*. Intrauterine infections, including herpes simplex virus, varicella-zoster virus, and rubella, may contribute to the pathogenesis of ACC. Furthermore, fetal exposure to certain teratogens, such as misoprostol, benzodiazepines, valproic acid, cocaine, methotrexate, angiotensin-converting enzyme (ACE) inhibitors, and methimazole, has been associated with an increased risk of ACC [5]. A summary of potential etiological factors contributing to the pathophysiology of ACC is provided in Table 1.

**Table 1:** Possible etiological factors contributing to the pathophysiology of aplasia cutis congenita (ACC) Source: [5]

Possible Cause	Examples
Multifactorial	Genetic and environmental
Trauma	Mechanical, physical, and vascular disruption
Amniotic fluid-related	Adhesions, amniotic band formation
Fetal	Vanishing twin, fetus papyraceus
Associated anomalies	Epidermolysis bullosa, ectodermal dysplasia, Adams-Oliver syndrome, Cutis marmorata, etc.
Genetic	Chromosomal abnormalities, gene
Environmental	Intrauterine infections, teratogens

Several classification systems exist for ACC, including the six-group system by Sybert and the nine-group system by Frieden [4]. Recent advancements in molecular genetics have led to a revised nine-group classification system, adapted from Frieden. The clinical presentation, including the location and appearance of the skin defect, can provide valuable clues to the presence of associated anomalies, which are incorporated into the classification systems. ACC is primarily a clinical diagnosis, with no specific histopathological findings. Previous case reports serve as valuable resources to guide the management of patients with this condition [6].

Management

Management of ACC typically involves wound care, including cleansing, dressing application, and the use of antibiotics. Conservative management is generally preferred for small lesions, with some reports demonstrating the successful healing of even extensive lesions using this approach. Surgical intervention may be necessary in certain cases. Early debridement and wound closure with split-thickness skin grafts, followed by subsequent replacement with a hairy scalp flap using tissue expansion techniques, is a common surgical approach. In some cases, a single application of a skin allograft has been used to cover extensive areas of skin loss. For more complex defects involving bone and soft tissue, composite reconstruction utilizing autologous full-thickness cranial bone grafts and scalp flaps may be required [7].

Both conservative and surgical approaches to managing ACC have inherent advantages and disadvantages. Conservative management offers several benefits, including ease of accessibility, suitability for management by pediatricians and dermatologists, and the avoidance of surgical risks. This approach is generally considered preferable for small, superficial lesions. However, conservative management may result in prolonged healing times and may be less effective in achieving the complete closure of larger, deeper lesions. Potential complications of conservative management include fluid loss from the wound, which can lead to electrolyte imbalances, hemorrhage, and an increased risk of infection [8].

Surgical intervention may become necessary if complications arise during conservative management. Surgical approaches are generally considered more suitable for achieving rapid closure of larger, deeper, and more extensive lesions. Potential complications associated with surgical management include donor site morbidity, flap necrosis, and graft rejection. The successful management of surgical interventions often requires the expertise and involvement of specialized surgical teams [9]. Table 2 presents the advantages and disadvantages of various options available for ACC treatment.

**Table 2:** Advantages and disadvantages of aplasia cutis congenita treatment Source: [8,9]

Conservative Treatment	Surgical Treatment
Advantages: Not invasive, takes care of almost all superficial lesions, lower cost, more comfortable, preferred for young and sensitive patients, no expert care is needed	Advantages: Better for severe, large, deep lesions or those not healing naturally, faster cure, better long-term prognosis
Disadvantages: May take a longer time, risk of infection, hemorrhage, delayed healing, and scar formation	Disadvantages: Invasive, higher cost, surgical complications, graft rejection, need of expert care

Our patient was diagnosed with 19q13.11 deletion syndrome, a condition characterized by a spectrum of clinical features, including poor growth, developmental delay, intellectual disability, dysmorphic features, cardiac abnormalities, short stature, ectodermal dysplasia, and hand and foot anomalies. 19q13.11 deletion syndrome was identified through the use of wide-genome screening techniques. Previous case reports have highlighted a correlation between the haploinsufficiency of the *UBA2* gene and the presence of ACC, emphasizing its significance as a clinical hallmark of this syndrome. This case shares overlapping features with syndromes involving 19q13.11 deletions [10].

A mutation in the *BMS1* is identified in ACC that affects ribosomal function, causing cell cycle and skin fibroblast defects, resulting in reduced cell proliferation. Thus, mutations in *BMS1* can affect the formation of a highly proliferative tissue during development, such as the rapidly expanding scalp epidermis. 19q13.11 deletion syndrome and the *UBA2* gene were associated with ACC and ectrodactyly as a specific feature in one of the studies. It can be assumed that this study conforms to the ACC with ectrodactyly skeletal syndrome (ACCES), a well-known genodermatosis linked to the *UBA2* gene [11].

Previous reports have described a phenotypic pattern of 19q13.11 deletion syndrome characterized by microcephaly, genital malformations in males (including hypospadias), ectodermal dysplasia, and developmental delay. 19q13.11 deletions and the Wilms tumor interacting protein (*WTIP*) gene result in hypospadias, making it a possible reason for this genital abnormality due to its well-known interaction with *WT1* [12]. The genetic abnormality found in this patient highlights the importance of genetic testing in patients with ACC.

## Conclusions

This case report highlights the importance of investigating and treating well-known and lesser-known congenital anomalies associated with ACC. It emphasizes the need to discuss the advantages and disadvantages of available treatment options and a multidisciplinary approach to managing patients with ACC. The chromosomal study done in this patient allowed for the identification of genetic contributors to ACC, drawing more attention to tailoring management strategies for ACC. Genetic counseling and testing may be more important if ACC is not an isolated anomaly. The findings of this case may not be directly generalizable to all cases of ACC, but the report highlights the importance of considering genetic and syndromic associations. The findings of this case report will help improve our understanding of ACC and enhance outcomes for affected individuals and their families.
